# Structural Features and Phylogenetic Implications of Four New Mitogenomes of Caliscelidae (Hemiptera: Fulgoromorpha)

**DOI:** 10.3390/ijms22031348

**Published:** 2021-01-29

**Authors:** Nian Gong, Lin Yang, Xiang-Sheng Chen

**Affiliations:** 1 Institute of Entomology, Guizhou University, Guiyang 550025, China; gongn0921@foxmail.com (N.G.); yanglin6626@163.com (L.Y.); 2 The Provincial Special Key Laboratory for Development and Utilization of Insect Resources, Guizhou University, Guiyang 550025, China; 3 The Provincial Key Laboratory for Agricultural Pest Management of Mountainous Regions, Guizhou University, Guiyang 550025, China

**Keywords:** planthopper, Caliscelidae, mitogenome, phylogenetics, genomics

## Abstract

To explore the differences in mitogenome variation and phylogenetics among lineages of the Hemiptera superfamily Fulgoroidea, we sequenced four new mitogenomes of Caliscelidae: two species of the genus *Bambusicaliscelis* (Caliscelinae: Caliscelini), namely *Bambusicaliscelis flavus* and *B. fanjingensis*, and two species of the genus *Youtuus* (Ommatidiotinae: Augilini), namely *Youtuus strigatus* and *Y. erythrus*. The four mitogenomes were 15,922–16,640 bp (base pair) in length, with 37 mitochondrial genes and an AT-rich region. Gene content and arrangement were similar to those of most other sequenced hexapod mitogenomes. All protein-coding genes (PCGs) started with a canonical ATN or GTG and ended with TAA or an incomplete stop codon single T. Except for two transfer RNAs (tRNAs; trnS1 and trnV) lacking a dihydrouridine arm in the four species and trnC lacking a dihydrouridine stem in the *Youtuus* species, the remaining tRNAs could fold into canonical cloverleaf secondary structures. Phylogenetic analyses based on sequence data of 13 PCGs in the 28 Fulgoroidea species and two outgroups revealed that Delphacidae was monophyletic with strong support. Our data suggest that Fulgoridae is more ancient than Achilidae. Furthermore, Flatidae, Issidae, and Ricaniidae always cluster to form a sister group to Caliscelidae.

## 1. Introduction

Caliscelidae is a relatively small family within the large and diverse planthopper superfamily Fulgoroidea that comprises approximately 240 described species from 77 genera divided into five tribes (Caliscelini, Peltonotellini, Adenissini, Ommatidiotini, and Augilini) and two subfamilies (Ommatidiotinae and Caliscelinae) [1,2]. These species are extensively distributed worldwide. The genus *Bambusicaliscelis* was established by Chen and Zhang (2011) [3] and belongs to the tribe Caliscelini of the subfamily Caliscelinae. The genus *Youtuus* was established by Gong and Chen (2018) [4] and belongs to the tribe Augilini of the subfamily Ommatidiotinae. These two genera are restrictively distributed in southern China and primarily inhabit bamboo. They directly injure bamboo using their sucking and piercing mouthparts and indirectly damage bamboo by transmitting plant pathogens; this severely hampers bamboo production.

To date, a total of 13,774 species from 2465 genera in 33 families of Fulgoroidea have been recorded worldwide [2]. Despite their great diversity, no more than 30 complete or near-complete mitogenomes have been reported from six planthopper families. Further, the amount of data recorded about these families follow the order Delphacidae > Achilidae > Fulgoridae > Ricaniidae/Issidae > Flatidae [5]. Overall, the data distribution is extremely unbalanced, and mitogenomes have not been reported in some families, whereas for others, there are no recorded molecular data. At present, most studies still rely on morphology for identifying and describing Caliscelidae, and few studies have discussed the taxonomic status of Caliscelidae within the superfamily Fulgoroidea based on molecular fragments (i.e., 18S, 28S, histone 3, and wingless) [6,7]. However, there is no available information about the complete mitogenomes of caliscelids, hindering molecular phylogenetic analyses among the Caliscelidae genera and species using molecular data. Moreover, the phylogenetic relationship between Caliscelidae and other planthopper families is limited. Therefore, better knowledge of the phylogenetic relationships among the members of Caliscelidae may be obtained by sequencing their mitogenomes. Further, additional information about taxa and more data are required to obtain a more completed and supported phylogeny of the Fulgoroidea superfamily in the future.

The insect mitogenome is a compact, double-stranded, closed circular molecule [8] containing 37 genes encoding 13 protein-coding genes (PCGs), 22 transfer RNAs (tRNAs), two ribosomal RNAs (rRNAs; *rrnS* and *rrnL*), and a noncoding AT-rich region [8,9]. Due to the small size of mtDNA as well as maternal inheritance, conserved gene content, a relatively fast evolutionary rate, and evolutionarily low levels of recombination, mitogenome sequences are the most widely used molecular tools for insect species identification and are becoming increasingly important for phylogenetic and evolutionary analyses of insects at the family level [10,11,12,13,14,15,16].

In the present study, we sequenced and annotated the complete mitogenomes of four Caliscelidae species, two from *Bambusicaliscelis* and two from *Youtuus*: *Bambusicaliscelis flavus*, *B. fanjingensis*, *Youtuus strigatus*, and *Y. erythrus*. Further, we analyzed the characteristics of these mitogenomes, including nucleotide composition, tRNA secondary structure, codon usage, gene overlaps, and noncoding regions, and compared the genetic distances between Caliscelidae and six other reported planthopper families. Comparative mitogenomics of these new sequences will provide a preliminary understanding of the structures and basic characteristics of Caliscelidae mitogenomes. Moreover, phylogenetic analyses using these mitogenomes can reveal the phylogenetic position of Caliscelidae and shed light on its phylogenetic relationships with other families within Fulgoroidea. Overall, the molecular data obtained in this study can improve our understanding of the phylogeny and evolution of Fulgoroidea.

## 2. Materials and Methods

### 2.1. Species Collection and Taxonomic Identification

Specimens of *Y. strigatus*, *Y. erythrus*, and *B. fanjingensis* were collected from Guizhou Province in July 2018, and those of *B. flavus* were collected from Yunnan Province in August 2018. All fresh specimens were immediately preserved in absolute ethanol and then stored at −20 °C at the Institute of Entomology, Guizhou University, Guiyang, China. These four species were identified based on their morphological characteristics [3,4,17]; species identification was performed by Nian Gong.

### 2.2. DNA Extraction and Sequencing

Total genomic DNA was extracted from the muscle tissue of each Caliscelidae species using the Takara Genome DNA Extraction Kit (Sangon Biotech, Shanghai, China) according to the manufacturer’s instructions. Extracted DNA samples were stored at −20 °C. Mitogenomes were sequenced using a next-generation sequencing platform with Illumina Hiseq 2500 at OriGene (Beijing, China). Following quantification of the total DNA that was extracted, an Illumina TruSeq library with single species was generated from the pooled genomic DNA, with an average insert size of 480 bp. This library was sequenced on a full run of Illumina Hiseq 2500 with 500 cycles and paired-end sequencing (250 bp reads). Raw sequence data reported in this paper have been deposited in the Genome Sequence Archive in the BIG Data Center, Chinese Academy of Sciences, under accession codes CRA003721.

### 2.3. Genome Assembly, Annotation, and Analysis

The quality of the raw sequences was evaluated using FastQC v0.11.4 (www.bioinformatics.babraham.ac.uk/projects/fastqc). Putative mitogenome reads with an average quality value of <Q30 were removed before assembly. Then, the clean sequences were assembled using MitoZ v2.4 software [18] with default parameters and the mitogenome of *Ricania speculum* (Fulgoroidea: Ricaniidae; NC_031369) as a reference.

The four mitogenomes were initially annotated using the MITOS web server (http://mitos.bioinf.uni-leipzig.de/index.py) [19] with invertebrate genetic codes. PCGs were predicted by determining theirs open reading frames using the invertebrate mitochondrial genetic codon. rRNA genes and the AT-rich regions were determined by comparisons with the homologous sequences of other planthoppers from GenBank (GenBank accession numbers: MH324927–MH324931). The locations and secondary structures of tRNA genes were predicted using the tRNAscan-SE search server (http://lowelab.ucsc. edu/tRNAscan-SE) and the MITOS web server (http://mitos.bioinf.uni-leipzig.de/index.py) with invertebrate codon predictors [20]. Mitogenomic circular maps were depicted and annotated using Geneious R9 [21].

Nucleotide composition and relative synonymous codon usage (RSCU) were calculated using PhyloSuite v1.2.2 [22], and RSCU figures were drawn using the fmsb package [23] of R 3.6.1 [24]. Strand asymmetry was calculated according to the following formulas [25]: AT skew = (A − T)/(A + T) and GC skew = (G − C)/(G + C). Tandem repeats in the AT-rich region were searched using the Tandem Repeats Finder program (http://tandem.bu.edu/trf/trf.basic.submit.html) [26]. The overlapping regions and intergenic spacers between genes were manually counted. The sequence data of the four insect mitogenomes have been deposited to GenBank under the accession numbers MW281858–MW281861 for *B. flavus*, *B. fanjingensis*, *Y. strigatus*, and *Y. erythrus*, respectively.

### 2.4. Phylogenetic Analyses

In addition to the four mitogenomes obtained in this study, the complete mitogenomes of 24 planthopper species were downloaded from GenBank for phylogenetic analyses; the mitogenomes of *Maiestas dorsalis* and *Yanocephalus yanonis* were used as outgroups. Detailed information and accession numbers of these mitogenomes are listed in Table S1.

The nucleotide sequences of 13 PCGs were aligned using MEGA v7.0.26 [27] with the Muscle [28]. Individual gene alignments were concatenated into a dataset using SequenceMatrix v1.7 [29]: nucleotide sequences of the 13 PCGs. The best partition schemes and substitution models for maximum likelihood (ML) and Bayesian inference (BI) analyses of the dataset were estimated using PartitionFinder v2.1.1 [30] with the greedy algorithm. Details of the best-fit partitioning schemes for ML and BI analyses are listed in Table S2. ML analyses were performed using IQ-Tree v1.6.3 [31], with 1000 replicates of ultrafast-likelihood bootstraping [32] to obtain node support values. BI analysis was performed using MrBayes v3.2.6 [33] under the following conditions: independent substitution model for each partition, four Markov chains, two independent runs each for 1,000,000 generations, sampling every 1000th generation, and the first 25% discarded as burn-in. When the average standard deviation of split frequencies fell below 0.01 and remained stable, stationarity was considered to have reached. Furthermore, an effective sample size value of more than 200 was used as a convergence diagnostic. When the bootstrap percentage (BP) was >75% or the Bayesian posterior probability (BPP) was >0.9, the results were considered credible. Consensus trees were viewed and edited using FigTree v1.4.2 [34].

## 3. Results

### 3.1. Genome Organization and Composition

**Figure 1:**
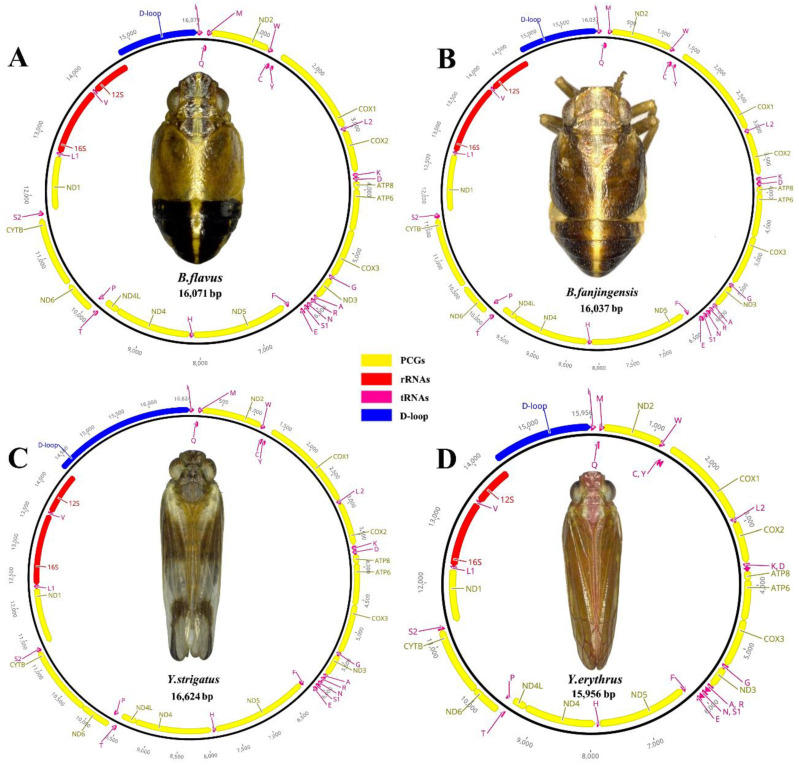
Circular maps of the mitogenomes of *Bambusicaliscelis flavus* (**A**), *B. fanjingensis* (**B**), *Youtuus strigatus* (**C**), and *Y. erythrus* (**D**). Protein-coding, ribosomal, and transfer RNA genes are shown with standard abbreviations. Gene orientations are indicated by arrow directions. Protein-coding genes are denoted in yellow, transfer RNA genes in aubergine, the AT-rich region in blue, and the two ribosomal RNA genes in red. The PCGs and rRNAs are the standard abbreviations. Each tRNA is denoted as a one-letter symbol according to the IUPAC-IUB single-letter amino acid codes.

The complete mitogenomes of *B. flavus* (GenBank No.: MW281858; length: 16,053 bp), *B. fanjingensis* (GenBank No.: MW281859; length: 15,979 bp), *Y. strigatus* (GenBank No.: MW281860; length: 16,640 bp), and *Y. erythrus* (GenBank No.: MW281861; length: 15,922 bp) were identified as circular double-stranded molecules (Figure 1). They were of medium size compared to those of other species of the Fulgoroidea superfamily. Each newly sequenced mitogenome contained 37 typical mitochondrial genes (13 PCGs, 2 rRNAs, and 22 tRNAs) and an AT-rich region (Figure 1; Table 1). All four caliscelid planthoppers displayed an identical gene arrangement that was shared with the putative ancestral gene order [35,36]. Of the typical genes, 23, including 9 PCGs and 14 tRNAs, were encoded on the major strand (J-strand), whereas the remaining 14 genes, including 4 PCGs, 8 tRNAs, and 2 rRNAs, were encoded on the minor strand (N-strand).

The nucleotide compositions and skewness levels of the newly obtained mitogenomes are provided in Table 2. The mean AT nucleotide content of the four mitogenomes was similar: 79.5% in *B. flavus*, 80.4% in *B. fanjingensis*, 79.8% in *Y. strigatus*, and 79.7% in *Y. erythrus*; these results indicate considerable AT bias. The AT-rich region had the highest AT content (82.3–89.1%), whereas PCGs had the lowest AT content (78.4–79.1%); these results are consistent with those of all previously sequenced planthopper mitogenomes [5]. Composition analysis of the new mitogenomes revealed that the whole mitogenomes exhibited positive AT skew (0.164–0.197), indicating that the presence of A was more than that of T, and negative GC skew (−0.293 to −0.238), indicating that the presence of C was more than that of G; these results correspond well with the AT bias observed in other Fulgoroidea mitogenomes.

### 3.2. PCGs and Codon Usage

The mitogenomes of all four planthoppers contained 13 PCGs. The concatenated lengths of the 13 PCGs in *B. flavus*, *B. fanjingensis*, *Y. strigatus*, and *Y. erythrus* were 11,039, 10,942, 10,933, and 10,901 bp, respectively; their respective average AT content was 78.4%, 78.8%, 79.1%, and 79%, respectively. The AT (−0.135 to −0.11) and GC (−0.062 to −0.042) skewness values of PCGs were similar among the four planthoppers (Table 2). Among the 13 PCGs, the nad5 gene was the largest and the atp8 gene was the smallest. Only four genes (nad1, nad4, nad4L, and nad5) were present on the minor strand, whereas the other nine PCGs (cox1, cox2, cox3, atp6, atp8, nad2, nad3, nad6, and cytb) were present on the major strand (Table 1; Figure 1).

The start and stop codons of all PCGs are listed in Table 1. In the four newly sequenced mitogenomes, most of the PCGs started with the typical ATN (ATA/T/G/C) codon, except for nad5 of *B. flavus*, *B. fanjingensis*, and *Y. strigatus*, which started with GTG. The complete termination codon TAA was more frequently used than TAG, whereas a single T was also used as the stop codon. The presence of an incomplete stop codon is common in insects [37], with reports suggesting that such a stop codon can be completed by post-transcriptional polyadenylation [10,38,39].

**Figure 2:**
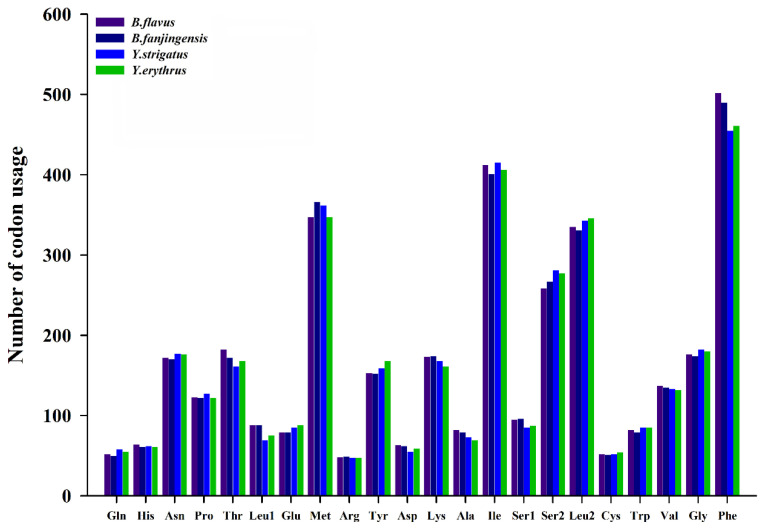
Number of codon usages in the protein-coding genes of *Bambusicaliscelis flavus*, *B. fanjingensis*, *Youtuus strigatus*, and *Y. erythrus* mitogenomes. Each amino acid is denoted as a three-letter symbol according to the IUPAC-IUB three-letter amino acid codes.

**Figure 3:**
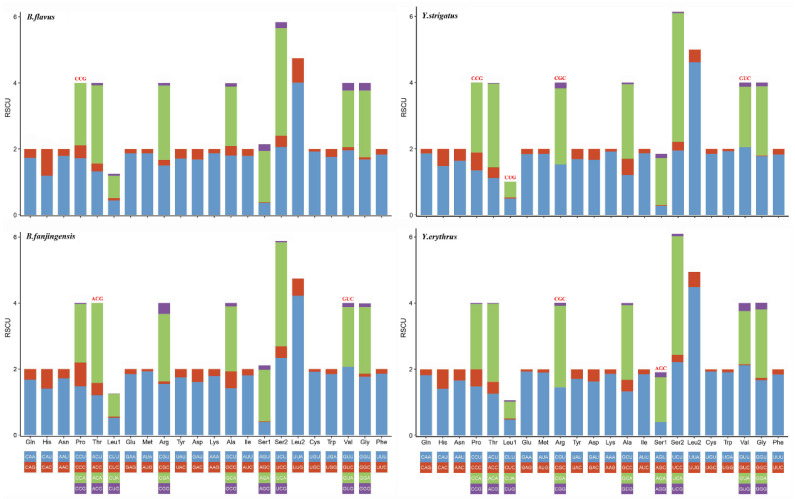
Relative synonymous codon usage in the protein-coding genes of *Bambusicaliscelis flavus*, *B. fanjingensis*, *Youtuus strigatus*, and *Y. erythrus* mitogenomes. Codon families are indicated below the X-axis. The color of the codon family below the X-axis corresponds to the color above the X-axis. The stop codon is not given. Codons absent in mitochondrial genomes are shown at the top of columns.

We determined the codon usage pattern, RSCU, and number in the PCGs of the four planthopper mitogenomes (Figure 2 and Figure 3; Table S3). The results demonstrated that codon usage was similar among the four species. The four most prevalent codons were Phe (UUU), Ile (AUU), Leu2 (UUA), and Met (AUA), accounting for more than half of the total number of amino acids. They all solely contained A or U, indicating high AT content in PCGs. The RSCU values of the PCGs indicated a pattern toward more A and T than G and C. In addition, codon usage showed an extremely high AT bias, which plays a key role in the AT bias of the whole mitogenome. All AT skew and GC skew of these PCGs were negative.

### 3.3. Ribosomal RNA and Transfer RNA Genes

In the complete mitogenomes of the four species, two rRNA genes (rrnL and rrnS) were encoded on the minor strand, with the second-highest AT content (>80%), positive GC skew (0.278−0.328), and negative AT skew (−0.203 to −0.157) (Table 2). The lengths of rrnL ranged from 1221 bp (*B. fanjingensis*) to 1227 bp (*B. flavus*), whereas those of rrnS ranged from 717 bp (*B. flavus*) to 738 bp (*B. fanjingensis*). Similar to other sequenced planthopper mitogenomes, rrnL was located between trnL2 and trnV, whereas rrnS was located between trnV and the AT-rich region.

All the 22 tRNA genes of the four mitogenomes were identified, with their total length ranging from 1406 to 1478 bp. The length of each tRNA was 56–74 bp and interspersed throughout the entire mitogenome. Among the 22 tRNA genes, 14 were encoded by the major strand and the remaining 8 were encoded by the minor strand, with a high AT content (>79%) and positive AT and GC skew. Except for trnS1 and trnV in the four species and trnC in the *Youtuus* species, which lacked a stable dihydrouridine (DHU) stem and was replaced by a simple loop, the remaining tRNA genes in the four new mitogenomes could be folded into the canonical cloverleaf secondary structure (Figures S1−S4). All the anticodons of the 22 tRNA genes are known to be identical across Hemiptera [5]. According to the predicted secondary structure, we identified a total of six mismatched base pairs, namely G-U, A-A, A-G, A-C, U-C, and U-U, as well as an extra A nucleotide. The total number of mismatched base pairs detected in the four planthoppers was 31 in *B. flavus*, 28 in *B. fanjingensis*, 32 in *Y. strigatus*, and 35 in *Y. erythrus*.

### 3.4. Overlapping and Intergenic Spacer Regions

We found 13–22 intergenic spacers in the four mitogenomes, which ranged in size from 1 to 58 bp. The longest (58 bp) intergenic spacer was identified in *B. fanjingensis*, between the trnF and nad5 genes. The four planthoppers had 4–10 overlapping genes, with overlaps ranging in size from 1 to 8 bp (Table 1). Two gene overlaps were conserved among the four newly sequenced mitogenomes: trnW–trnC (8 bp: AAGCCTTA) and atp8–atp6 (7 bp: ATGATAA (in *Youtuus*) or ATAATAA (in *Bambusicaliscelis*. A 7 bp overlapping region (TTAACAT (in *Bambusicaliscelis*) or TTAGCAT (in *Youtuus*)) located between nad4 and nad4L was detected in three of the four mitogenomes (excluding *Y. erythrus*). Further, an 8 bp overlapping region (ATGTTTAA) located between nad6 and cytb was detected in both the *Youtuus* species but not in either of the *Bambusicaliscelis* species.

### 3.5. AT-Rich Region

The AT-rich region is believed to be involved in regulating the replication and transcription of mtDNA in insects; in the four planthoppers, it was located between rrnS and trnI. Among the four sequenced mitogenomes of Caliscelidae, the lengths of the AT-rich regions considerably differed in the four mitogenomes, ranging from 1230 bp in *B. fanjingensis* to 2288 bp in *Y. strigatus*. Nevertheless, length differences among the mitogenomes were consistent with differences in their total length. The AT-rich region had the highest AT content, ranging from 82.3% in *Y. strigatus* to 89.1% in *B. fanjingensis*, with positive AT skew (0.011−0.068) and negative GC skew (−0.405 to −0.169) (Table 2). The structural organization of the AT-rich regions in the four planthopper mitogenomes is illustrated in Figures S5−S8. Four repeat regions were present in *B. flavus*, three were present in *B. fanjingensis* and *Y. strigatus*, and two were present in *Y. erythrus*. The largest repeat unit was 217 bp, with two repeats in *B. flavus*.

### 3.6. Phylogenetic Relationships

**Figure 4:**
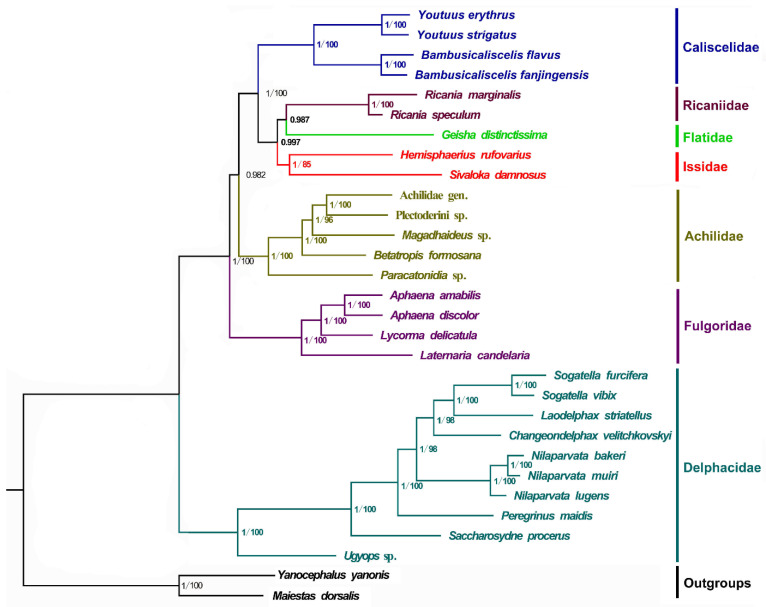
Phylogenetic trees of Fulgoroidea inferred using MrBayes (Bayesian inference) and maximum likelihood (ML) analysis based on the nucleotide sequences of the 13 protein-coding genes. Bayesian posterior probabilities (BPPs) and bootstrap percentage (BP) are indicated on branches; a BP below 75 is not shown.

Phylogenetic relationships among the 28 Fulgoroidea species (including the four newly sequenced mitogenomes) and two outgroup species were determined by concatenating the nucleotide sequences of the 13 PCGs using BI and ML analyses. A total of two phylogenetic trees (BI-PCG, and ML-PCG) were reconstructed using BI and ML analyses (Figure 4). In two trees, 10 Delphacidae species formed a steadily monophyletic group with high support (BPP = 1/BP = 100) and were located at the basal position of the phylogenetic tree. This finding is consistent with that of previous studies [5,40,41,42,43,44]. In all trees, four Fulgoridae species formed a cluster that was located near the clade of Delphacidae with high support (BPP = 1/BP = 100), whereas four families (Caliscelidae, Ricaniidae, Flatidae, and Issidae) gathered to form a sister group to Achilidae. Moreover, three families (Ricaniidae, Flatidae, and Issidae) assembled to form a sister group to Caliscelidae. The four Caliscelidae species always clustered with high support (BPP = 1/BP = 100).

## 4. Discussion

In the present study, we successfully sequenced and analyzed the complete mitogenomes of four Caliscelidae species. Among the four mitogenomes, the length ranged from 15,922 bp in *Y. erythrus* to 16,640 bp in *Y. strigatus*. The difference in length is mainly attributed to the difference in the size of the intergenic spacer regions and the length of the AT-rich region. The intergenic spacer regions of *Bambusicaliscelis* are longer than those of *Youtuus*, whereas the AT-rich region of *Youtuus* is longer than that of *Bambusicaliscelis*. Except for atp8 (37 bp), nad5 (59 bp), and nad4 (58 bp), the length differences of each PCG in the four newly sequenced mitogenomes did not exceed 23 bp; this result suggests that the PCGs have relatively conserved characteristics among species. However, the length variation of tRNAs and rRNAs among the different species was limited, which is mainly due to the stability of their secondary structures.

Among the secondary structures of the 22 putative tRNAs, trnS1 and trnV lacked a DHU stem and formed a simple loop in the four species. The DHU arm of trnS1 formed a simple loop, which has been recognized in many metazoans [45,46,47]; however, we are the first to report the lack of a DHU stem in trnV in Caliscelidae. We found that trnC also lacks a DHU stem and forms a simple loop in the two *Youtuus* species; this finding is in contrast to the normal structure observed in the two *Bambusicaliscelis* species and may be related to the evolution of these species. The genera *Bambusicaliscelis* and *Youtuus* are part of the tribes Caliscelini and Augilini, respectively, and the species in these two tribes differ considerably based on appearance. For instance, the species occurring in Augilini have hind wings, whereas those occurring in Caliscelini exhibit hind-wing degeneration. These are species-specific differences.

In many Fulgoroidea species, an 8 bp overlapping AAGCCTTA region between trnW and trnC has been detected [5]; the four newly sequenced mitogenomes of Caliscelidae also show this characteristic. Further, a 7 bp overlap between atp8 and atp6 was detected in other planthoppers. In the present study, analysis showed that the 7 bp overlapping region in *Bambusicaliscelis* is coded as ATAATAA and that in *Youtuus* is coded as ATGATAA. Perhaps, owing to differences between the genera, there is a A/G difference. An 8 bp overlapping region (ATGTTTAA) was located between nad6 and cytb in both the *Youtuus* species but not in either of the *Bambusicaliscelis* species; these are species-specific differences.

In the phylogenetic analysis, Delphacidae species formed a steady monophyletic group with high support (BPP = 1/BP = 100) and were located at the basal position of the phylogenetic tree, consistent with the findings of previous studies [5,40,41,42,43,44]. In contrast to the study of Xu et al. [5], who found Achilidae to be more ancient than Fulgoridae, our analysis indicated that Fulgoridae was more ancient than Achilidae; this discrepancy may be associated with the number of selected species and the different taxa used in each study. However, our result is consistent with the divergence time estimation of Fulgoroidea analyzed by Song and Liang, wherein the chronogram of Fulgoroidea showed that the lineage Achilidae could be traced back to the early Palaeocene era (46 Mya), whereas Fulgoridae diverged in the early Cretaceous era (approximately 78 Mya) [48]. In addition, we found that Flatidae, Issidae, and Ricaniidae always gathered to form a sister group to Caliscelidae. The placement of the Ricaniidae sister to Flatidae and Issidae was consistently supported in both analyses, with strong nodal supports (BPP > 0.98), consistent with the findings of morphological, nuclear genes and mitochondrial genes [6,40,41]. Because Issidae had only two effective mitogenome sequences, the paraphyletic of Issidae observed in other molecular phylogenetic analyses was not supported in this study [43]. The four Caliscelidae species always clustered with high support (BPP = 1/BP = 100). Although the representative mitogenomes from Fulgoroidea are still limited, the findings of phylogenetic analyses will be more reliable and convincing because information about more mitogenomes and genomes of Fulgoroidea species is available in databases.

## Figures and Tables

**Table 1:** List of total size and intergenic nucleotides for mitochondrial genes of *B. flavus, B. fanjingensis, Y. strigatus*, and *Y. erythrus*, with lengths of genes, anticodons of tRNAs, and start/stop codons of protein-coding genes.

Gene	*B. flavus*			*B. fanjingensis*			*Y. strigatus*			*Y. erythrus*		
	Size	Anticodons	Int.	Size	Anticodons	Int.	Size	Anticodons	Int.	Size	Anticodons	Int.
		Start/Stop			Start/Stop			Start/Stop			Start/Stop	
trnI	67	ATC		67	ATC		65	ATC		65	ATC	
*trnQ*	69	CAA	7	69	CAA	9	69	CAA	7	69	CAA	7
trnM	67	ATG	13	66	ATG	20	64	ATG	−1	64	ATG	−1
nad2	966	ATT/TAA	0	966	ATT/TAA	0	966	ATT/TAA	0	966	ATT/TAA	0
trnW	65	TGA	−1	63	TGA	0	68	TGA	−1	68	TGA	−1
*trnC*	74	TGC	−8	73	TGC	−8	57	TGC	−8	58	TGC	−8
*trnY*	67	TAC	12	66	TAC	15	63	TAC	4	62	TAC	6
cox1	1539	ATG/TAA	23	1536	ATG/TAA	23	1534	ATG/T	9	1534	ATG/T	10
trnL2	70	TTA	1	70	TTA	8	66	TTA	0	68	TTA	0
cox2	675	ATT/TAA	0	675	ATT/TAA	0	675	ATA/TAA	0	675	ATA/TAA	0
trnK	70	AAG	20	71	AAG	40	71	AAG	20	71	AAG	8
trnD	69	GAC	2	70	GAC	0	66	GAC	0	65	GAC	0
atp8	134	ATT/TAA	0	119	ATT/TAA	2	156	ATT/TAA	0	156	ATT/TAA	0
atp6	652	ATA/T	−7	655	ATA/T	−7	652	ATG/T	−7	652	ATG/T	−7
cox3	783	ATG/TAA	0	783	ATG/TAA	0	781	ATG/T	0	781	ATG/T	0
trnG	65	GGA	7	66	GGA	26	66	GGA	0	63	GGA	−1
nad3	348	ATA/TAA	0	348	ATA/TAA	0	348	ATT/TAG	0	348	ATT/TAA	−1
trnA	69	GCA	−1	68	GCA	6	64	GCA	−2	64	GCG	0
trnR	66	CGA	3	65	CGA	12	63	CGA	3	59	CGA	4
trnN	71	AAC	23	71	AAC	3	67	AAC	4	67	AAC	8
trnS1	60	AGC	−1	61	AGC	−1	59	AGC	−1	58	AGC	−1
trnE	67	GAA	18	71	GAA	10	62	GAA	−1	62	GAA	−1
*trnF*	65	TTC	37	62	TTC	34	63	TTC	0	64	TTC	0
*nad5*	1713	GTG/TAA	48	1671	GTG/TAA	58	1678	GTG/T	4	1654	ATG/T	3
*trnH*	72	CAC	0	70	CAC	0	60	CAC	0	62	CAC	0
*nad4*	1366	ATG/T	1	1321	ATG/T	1	1317	ATG/TAA	21	1308	ATA/TAG	27
*nad4l*	270	ATG/TAA	−7	270	ATG/TAA	−7	273	ATG/TAA	−7	273	ATG/TAA	1
trnT	69	ACA	2	69	ACA	38	67	ACA	2	67	ACA	2
*trnP*	63	CCA	4	66	CCA	26	63	CCA	9	66	CCA	4
nad6	504	ATA/TAA	1	501	ATA/TAA	1	492	ATA/TAA	1	492	ATA/TAA	1
cob	1140	ATG/TAA	32	1134	ATG/TAA	33	1119	ATG/TAA	−8	1117	ATG/T	−8
trnS2	68	TCA	48	70	TCA	11	62	TCA	2	64	TCA	0
*nad1*	949	ATG/T	−4	963	ATG/TAA	16	942	ATG/TAA	6	945	ATG/TAA	−1
*trnL1*	67	CTA	3	63	CTA	1	65	CTA	1	64	CTA	1
*rrnL*	1227	-	0	1221	-	0	1222	-	0	1222	-	0
*trnV*	56	GTA	0	61	GTA	0	58	GTA	0	56	GTA	0
*rrnS*	717	-	0	738	-	0	732	-	0	732	-	0
CR	1318		0	1230		0	2288		0	1609		0
Total	16053			15979			16640			15922		

Note: Except for the AT-rich region, the italicized genes were encoded by the H-strand, with others being encoded by the L-strand. CR: AT-rich region. Int: length of the intergenic spacer region, for which negative numbers indicate nucleotide overlapping between adjacent genes.

**Table 2:** Base composition of four mitochondrial whole genomes, 13 protein-coding genes (PCGs), 22 transfer RNAs (tRNAs), 2 ribosomal RNAs (rRNAs), and AT-rich regions.

Regions	Size (bp)	T(U)	C	A	G	A+T (%)	G+C (%)	AT Skewness	GC Skewness
Full Genome									
*B. flavus*	16053	32	13.2	47.5	7.2	79.5	20.4	0.194	−0.293
*B. fanjingensis*	15979	32.2	12.6	48.1	7	80.3	19.6	0.197	−0.288
*Y. strigatus*	16640	33	12.5	46.8	7.7	79.8	20.2	0.173	−0.238
*Y. erythrus*	15922	33.3	12.7	46.4	7.6	79.7	20.3	0.164	−0.251
13 PCGs									
*B. flavus*	11039	44.5	11.2	33.9	10.3	78.4	21.5	−0.135	−0.042
*B. fanjingensis*	10942	44.5	11.2	34.3	9.9	78.8	21.1	−0.129	−0.062
*Y. strigatus*	10933	43.9	11.1	35.2	9.8	79.1	20.9	−0.11	−0.062
*Y. erythrus*	10901	44.4	11.0	34.6	10.0	79	21	−0.124	−0.048
2 rRNAs									
*B. flavus*	1944	47.7	6.6	32.8	12.9	80.5	19.5	−0.185	0.323
*B. fanjingensis*	1959	46.7	6.5	34	12.8	80.7	19.3	−0.157	0.328
*Y. strigatus*	1954	48.7	6.7	32.2	12.4	80.9	19.1	−0.203	0.298
*Y. erythrus*	1954	47.5	6.9	33.5	12.1	81	19	−0.172	0.278
22 tRNAs									
*B. flavus*	1476	37.8	9.1	42.1	11.0	79.9	20.1	0.054	0.095
*B. fanjingensis*	1478	37.1	8.9	43.4	10.7	80.5	19.6	0.078	0.092
*Y. strigatus*	1408	37.4	8.8	42.0	11.8	79.4	20.6	0.058	0.146
*Y. erythrus*	1406	38.0	8.6	41.1	12.3	79.1	20.9	0.039	0.177
AT-rich regions									
*B. flavus*	1318	39.3	11.1	44.9	4.7	84.2	15.8	0.067	−0.405
*B. fanjingensis*	1230	41.5	7.4	47.6	3.5	89.1	10.9	0.068	−0.358
*Y. strigatus*	2288	38.8	10.3	43.5	7.3	82.3	17.7	0.057	−0.169
*Y. erythrus*	1609	40.9	11.5	41.8	5.8	82.7	17.3	0.011	−0.329

## Data Availability

Not applicable.

## Supplementary Materials

The following are available online at https://www.mdpi.com/1422-0067/22/3/1348/s1: Figure S1. Secondary structures of transfer RNAs (tRNAs) in the mitogenome of *Bambusicaliscelis flavus*. Dashes indicate Watson–Crick base pairing, and dots indicate GU base pairing; Figure S2. Secondary structures of transfer RNAs (tRNAs) in the mitogenome of *Bambusicaliscelis fanjingensis*. Dashes indicate Watson–Crick base pairing, and dots indicate GU base pairing; Figure S3. Secondary structures of transfer RNAs (tRNAs) in the mitogenome of *Youtuus strigatus*. Dashes indicate Watson–Crick base pairing, and dots indicate GU base pairing; Figure S4. Secondary structures of transfer RNAs (tRNAs) in the mitogenome of *Youtuus erythrus*. Dashes indicate Watson–Crick base pairing, and dots indicate GU base pairing; Figure S5. Features present in the AT-rich region of the *Bambusicaliscelis flavus* mitogenome. The four tandem repeats are shaded in yellow, gray, purple-red, and blue, respectively. * indicates a mismatch;—indicates an insertion or deletion; Figure S6. Features present in the AT-rich region of the *Bambusicaliscelis fanjingensis* mitogenome. The three tandem repeats are shaded in yellow, gray, and blue, respectively. * indicates a mismatch;—indicates an insertion or deletion; Figure S7. Features present in the AT-rich region of the *Youtuus strigatus* mitogenome. The three tandem repeats are shaded in yellow, gray, and blue, respectively. * indicates a mismatch;—indicates an insertion or deletion; Figure S8. Features present in the AT-rich region of the *Youtuus erythrus* mitogenome. The two tandem repeats are shaded in yellow and gray, respectively. * indicates a mismatch;—indicates an insertion or deletion; Table S1. Mitogenomes used in this study; Table S2. The partition schemes and best substitution models used in the phylogenetic analysis; Table S3. Codon number and relative synonymous codon usage (RSCU) in the four mitochondrial PCGs.
