# Supplementary figures and images for: Structural Features and Phylogenetic Implications of Four New Mitogenomes of Caliscelidae (Hemiptera: Fulgoromorpha)
Source: Int J Mol Sci. 2021 Jan 29;22(3):1348. doi: 10.3390/ijms22031348 (PMC7866285; doi:10.3390/ijms22031348)

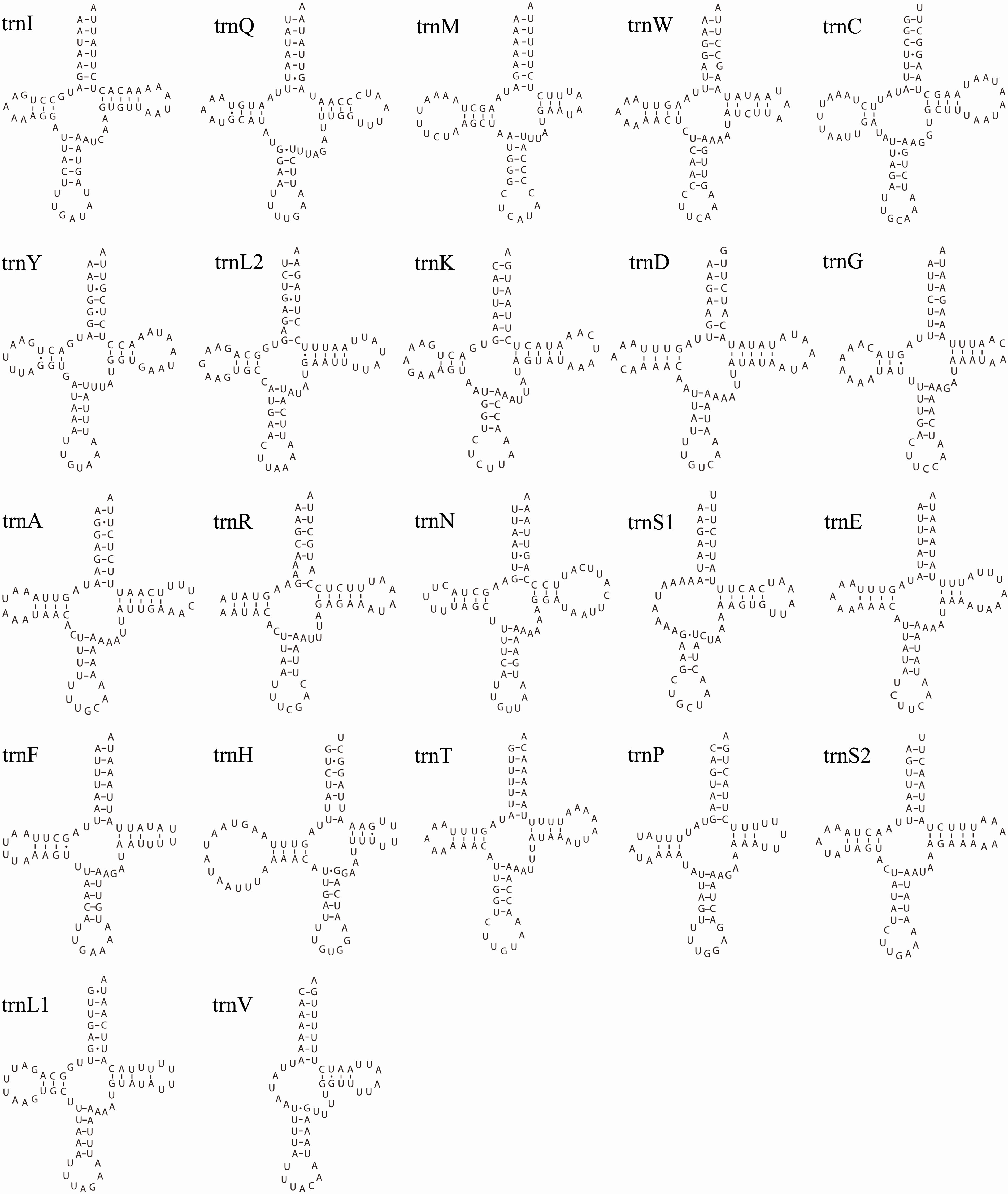

Supplement: Supplementary file 1 [file ijms-22-01348-s001.zip › supplementary materials/Figure S1 (B.flavus).jpg]

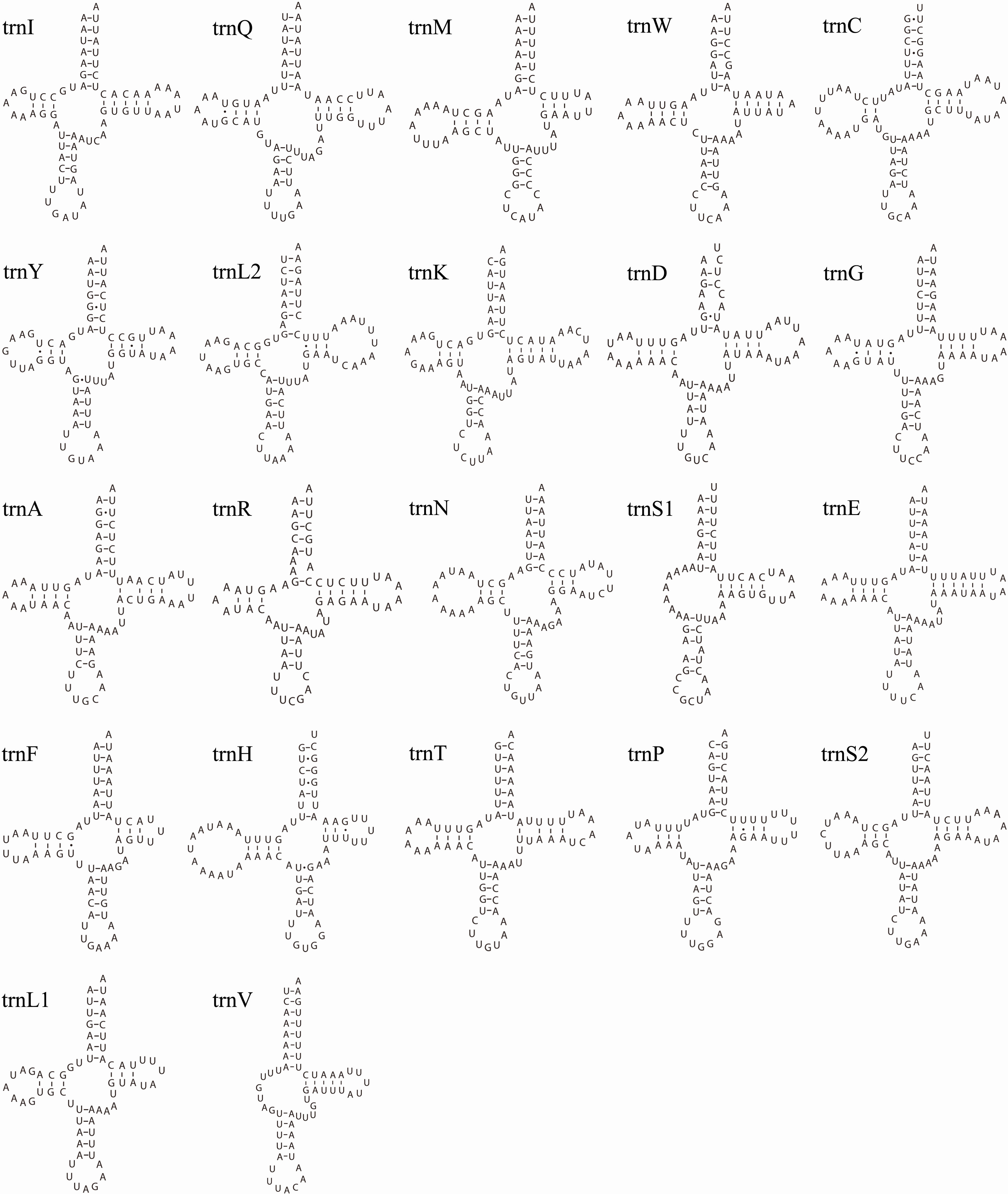

Supplement: Supplementary file 1 [file ijms-22-01348-s001.zip › supplementary materials/Figure S2 (B.fanjingensis).jpg]

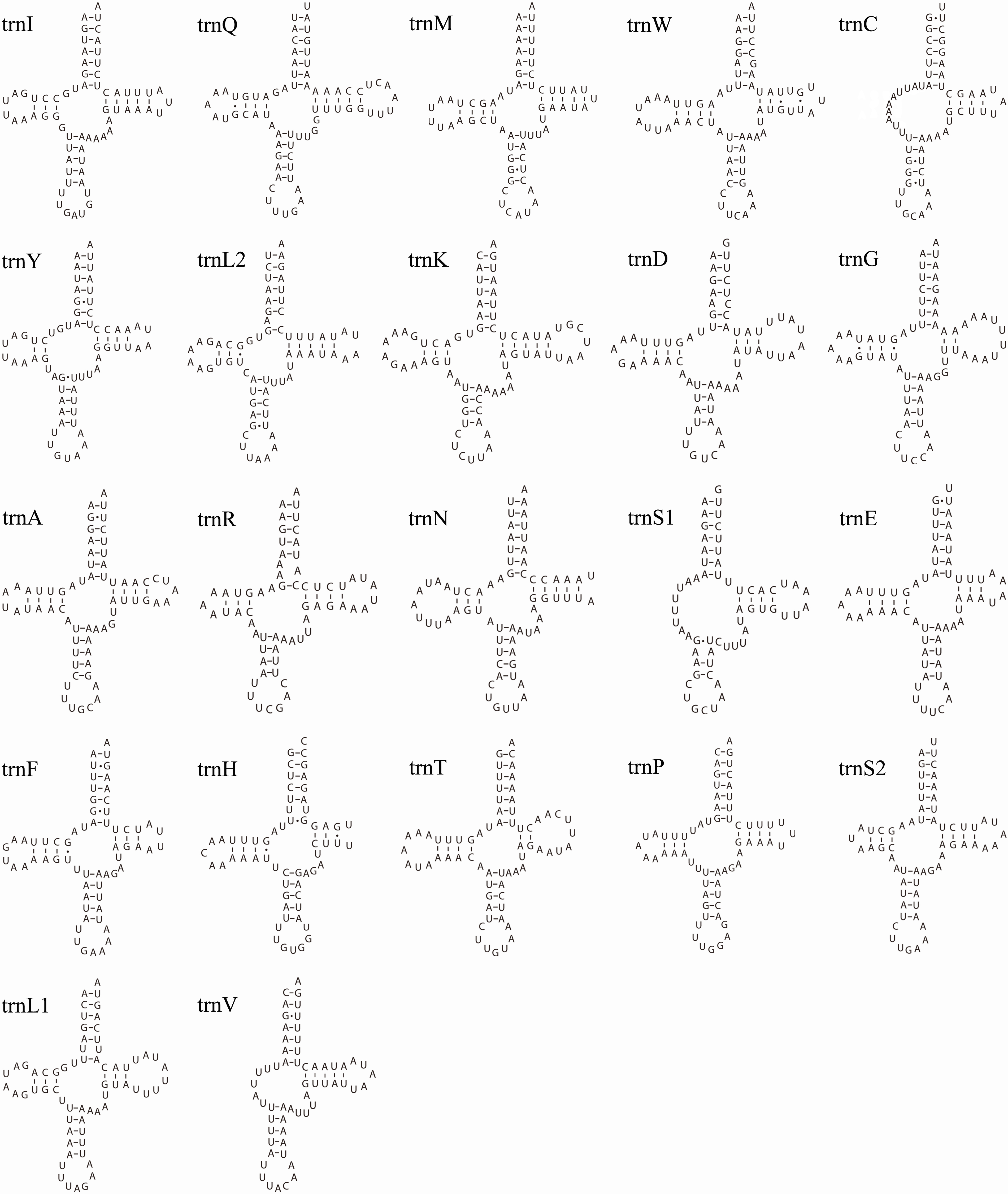

Supplement: Supplementary file 1 [file ijms-22-01348-s001.zip › supplementary materials/Figure S3 (Y.strigatus).jpg]

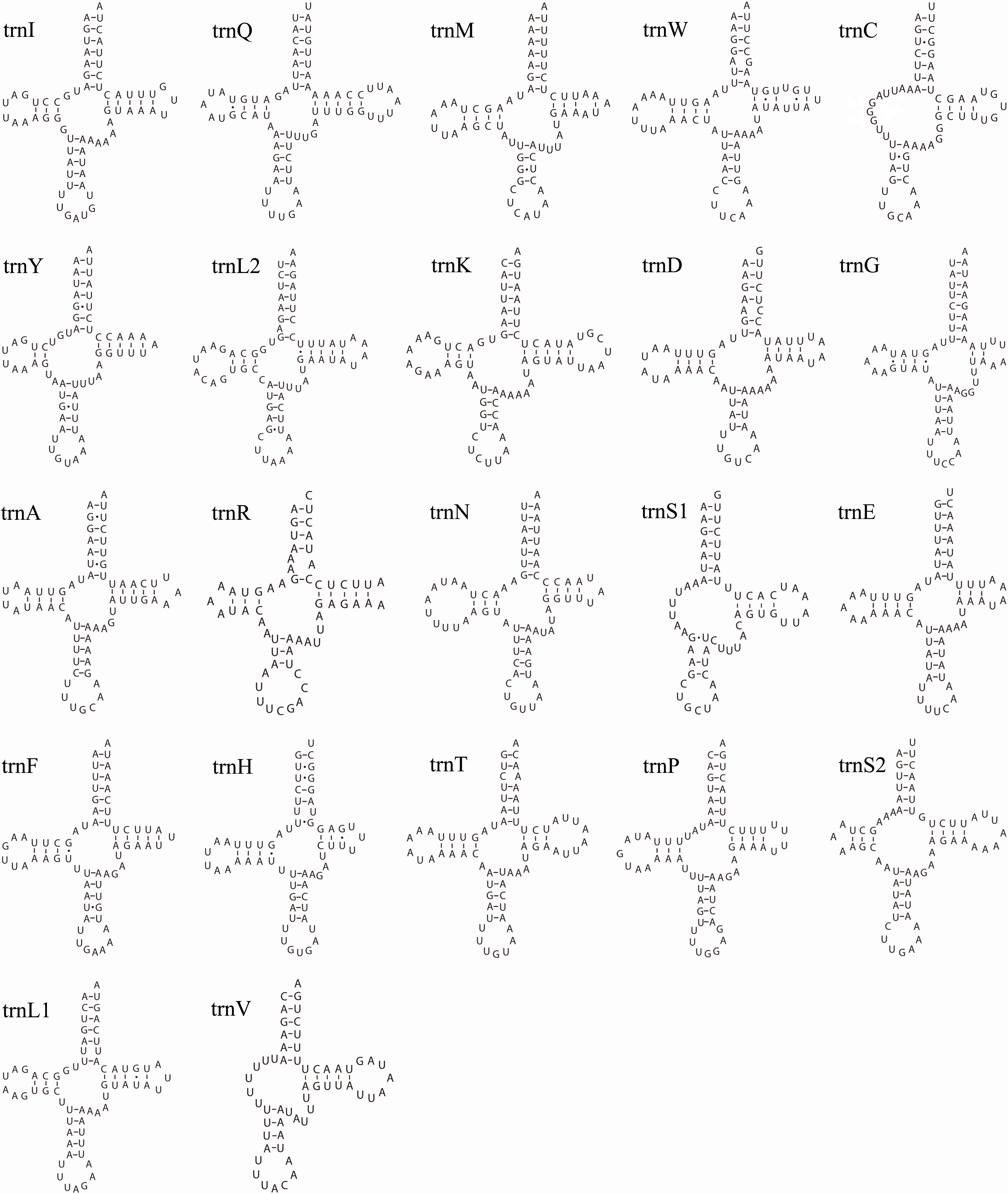

Supplement: Supplementary file 1 [file ijms-22-01348-s001.zip › supplementary materials/Figure S4 (Y.erythrus).jpg]

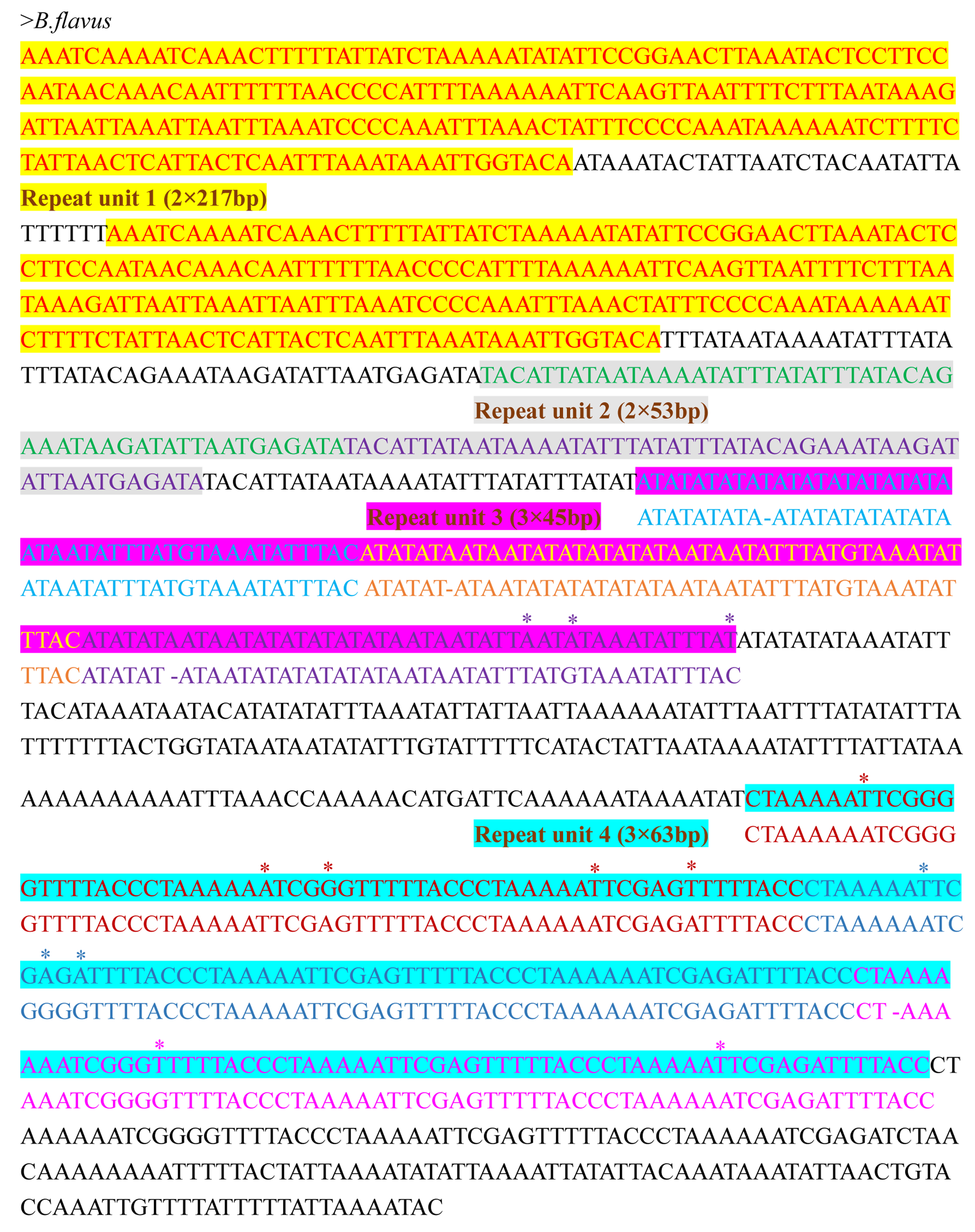

Supplement: Supplementary file 1 [file ijms-22-01348-s001.zip › supplementary materials/Figure S5 (B.flavus).jpg]

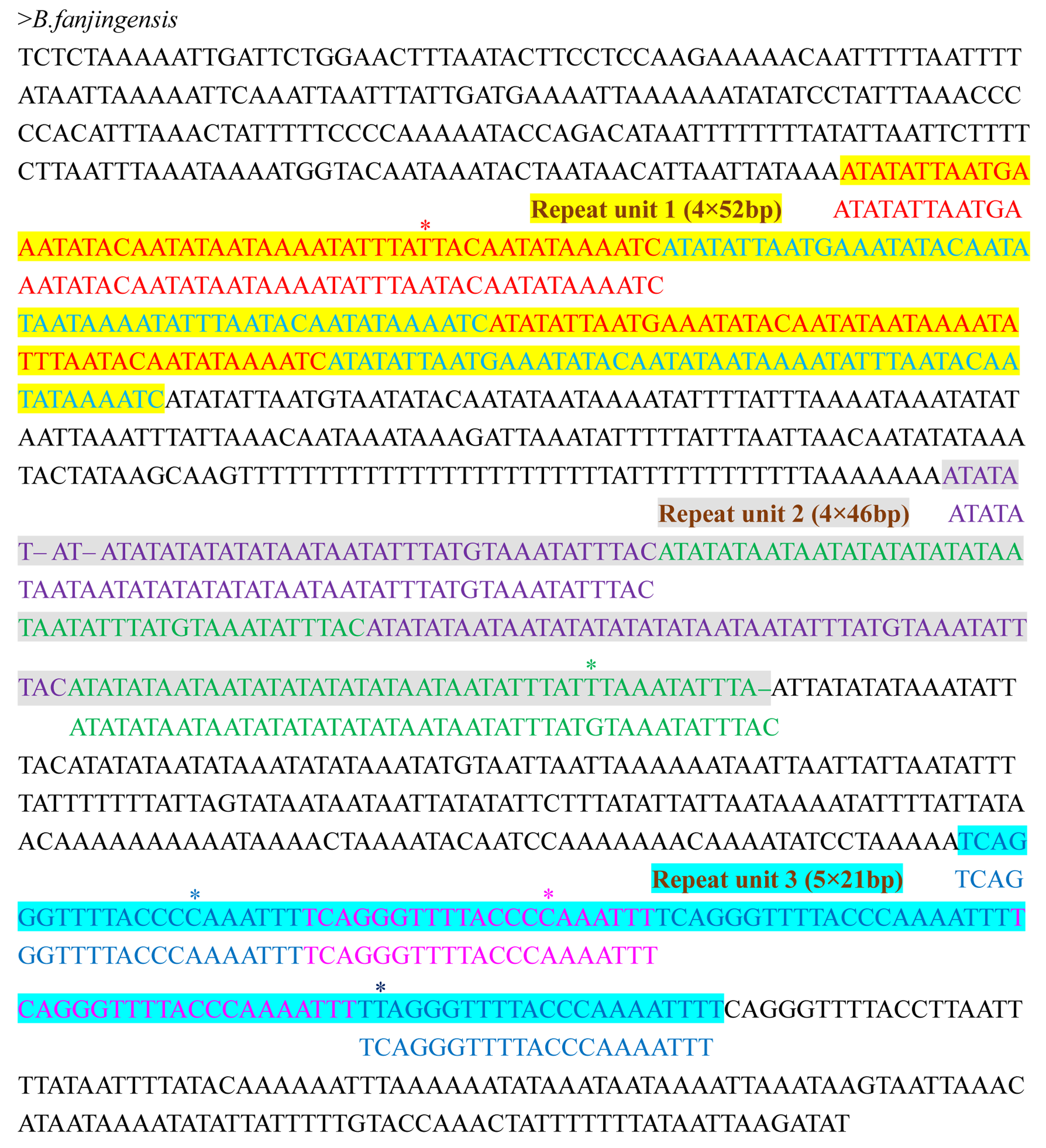

Supplement: Supplementary file 1 [file ijms-22-01348-s001.zip › supplementary materials/Figure S6 (B.fanjingensis).jpg]

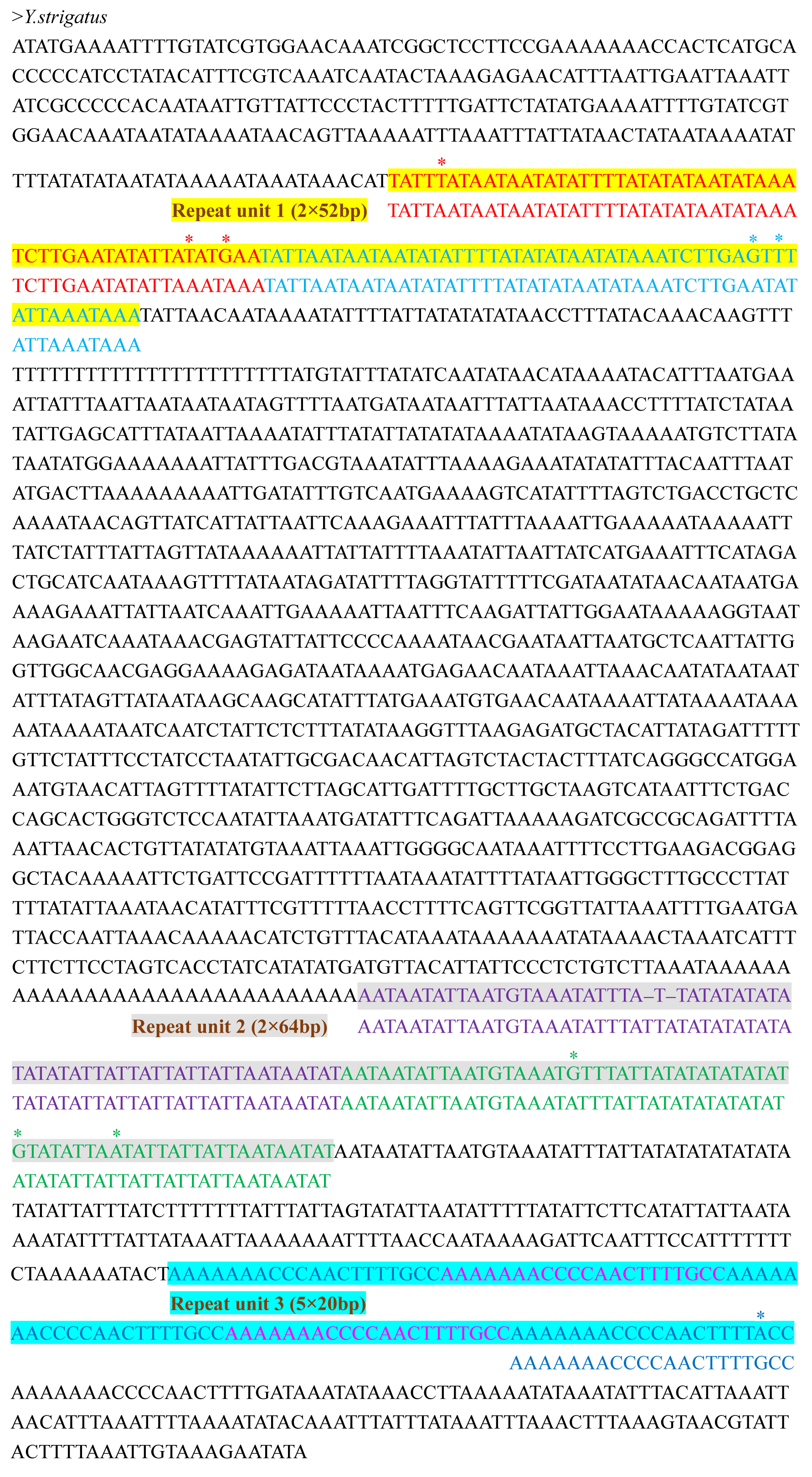

Supplement: Supplementary file 1 [file ijms-22-01348-s001.zip › supplementary materials/Figure S7 (Y.strigatus).jpg]

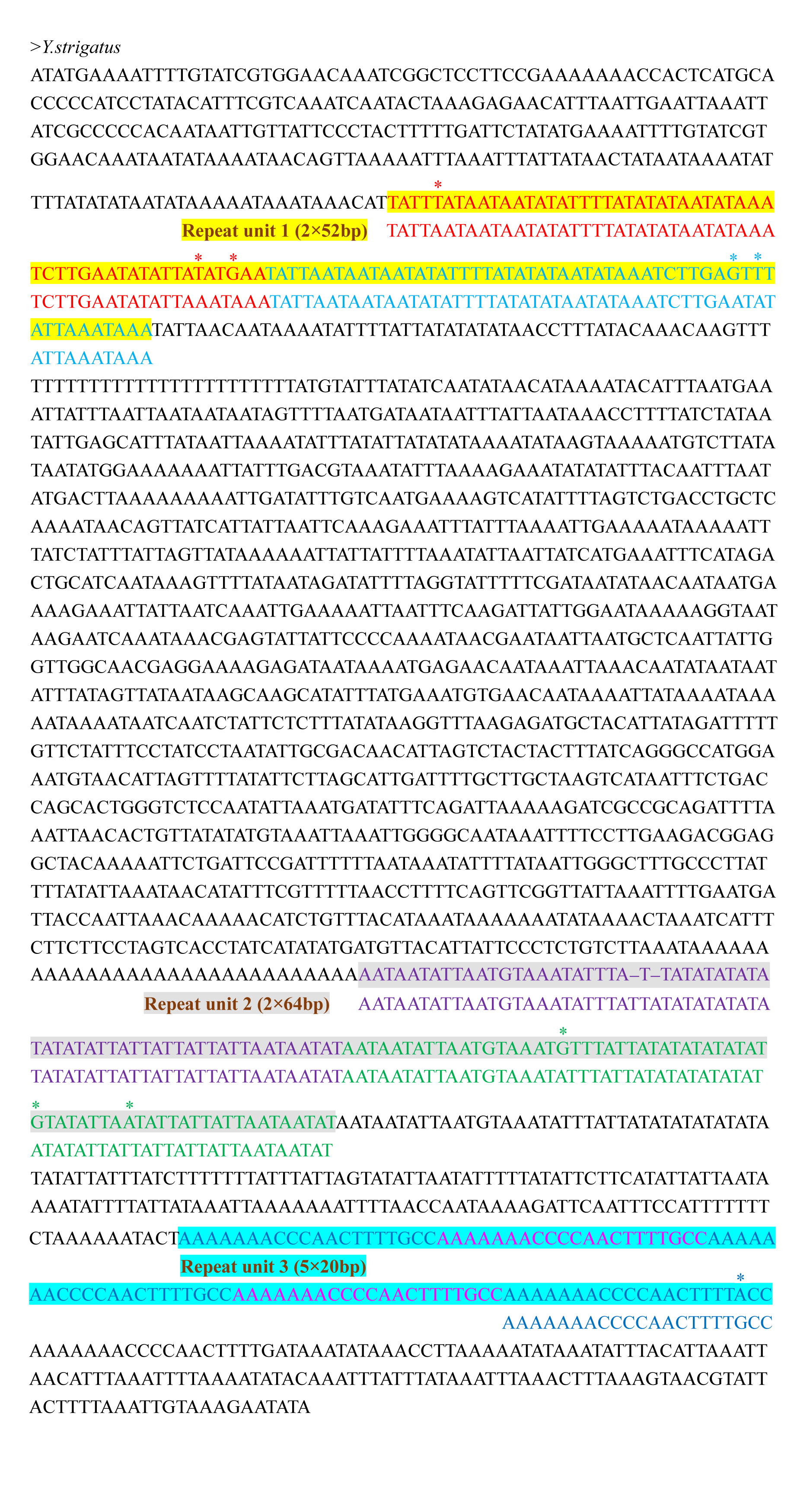

Supplement: Supplementary file 1 [file ijms-22-01348-s001.zip › supplementary materials/Figure S7 (Y.strigatus).tif]

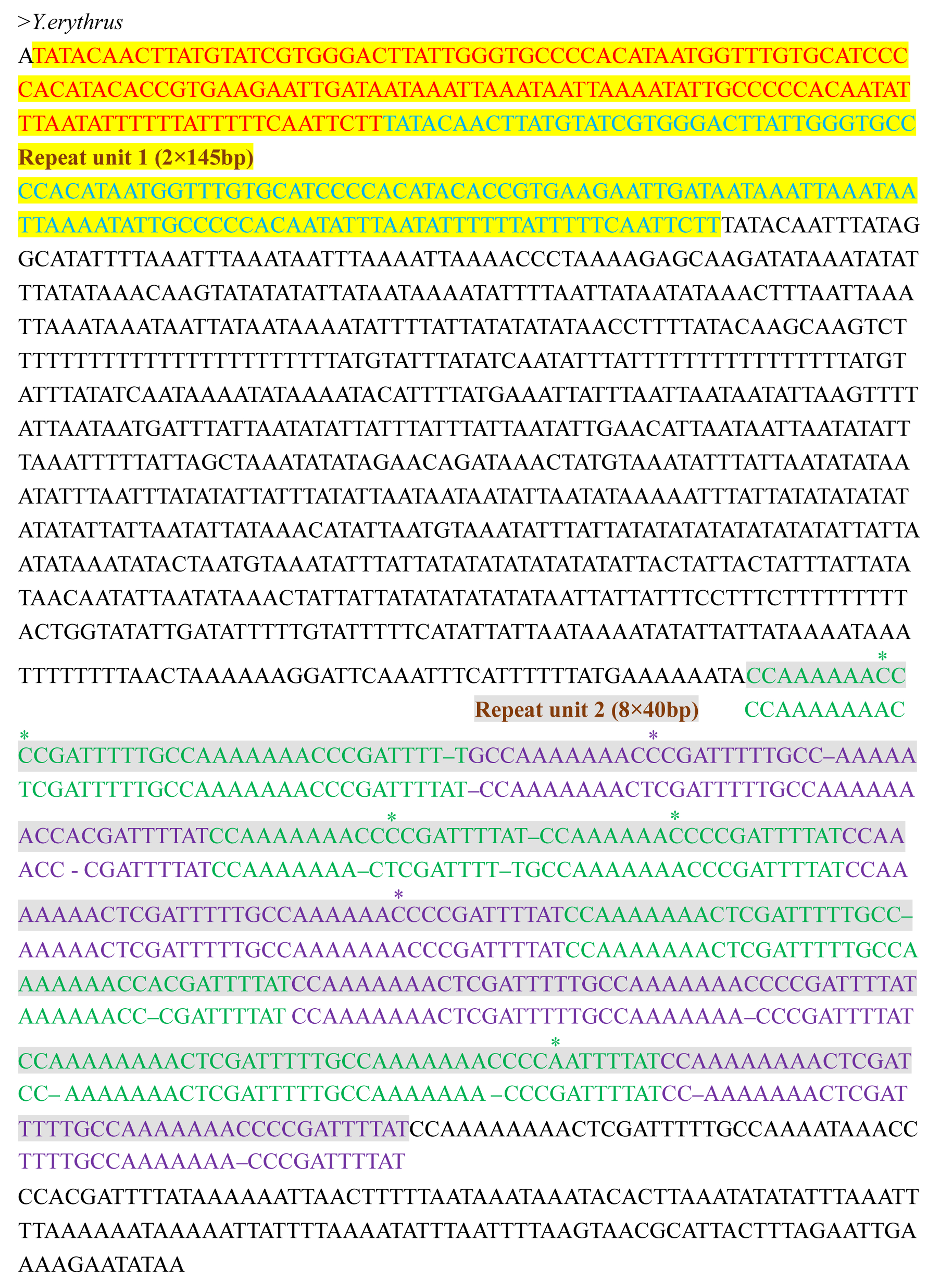

Supplement: Supplementary file 1 [file ijms-22-01348-s001.zip › supplementary materials/Figure S8 (Y.erythrus).jpg]

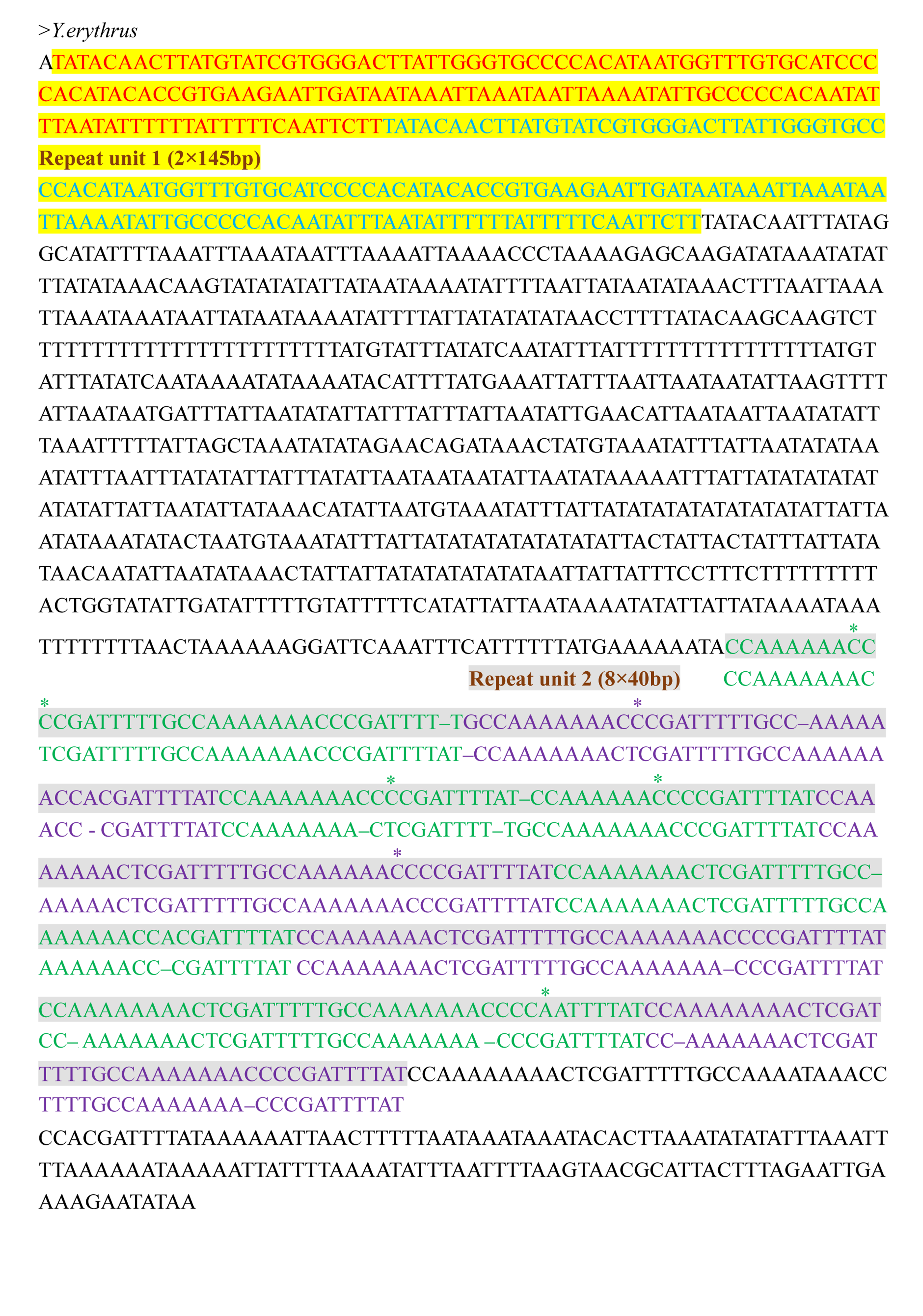

Supplement: Supplementary file 1 [file ijms-22-01348-s001.zip › supplementary materials/Figure S8 (Y.erythrus).tif]
